# Aging‐associated changes in immunological parameters: Implications for COVID‐19 immune response in the elderly

**DOI:** 10.14814/phy2.70364

**Published:** 2025-05-22

**Authors:** Maha Gasmi, Mahdi Hejazi, Antonella Muscella, Santo Marsigliante, Aastha Sharma

**Affiliations:** ^1^ Higher Institute of Sport and Physical Education of Ksar Said Tunis Tunisia; ^2^ Department of Nutrition, School of Public Health Iran University of Medical Sciences Tehran Iran; ^3^ Department of Biological and Environmental Science and Technologies (DiSTeBA) University of Salento Lecce Italy; ^4^ Department of Basic and Applied Science, School of Engineering and Science University‐GD Goenka University Gurugram Gurugram India

**Keywords:** aging, COVID‐19, elderly, immune health, SARS‐COV‐2

## Abstract

Aging has a profound impact on the immune system, leading to a gradual decline in its function and increased systemic inflammation, collectively known as immunosenescence and inflammaging. These changes make older adults more susceptible to infections, including COVID‐19, and contribute to worse clinical outcomes, such as higher morbidity and mortality rates. This review explores immunological changes associated with aging, including impaired innate immune responses, reduced T‐ and B‐cell function, and altered cytokine profiles. A comprehensive literature search identified relevant studies on the topic, and inclusion criteria focused on studies addressing age‐related immune changes and their impact on responses to COVID‐19. The findings underscore the need for targeted healthcare strategies to mitigate the negative effects of aging on immunity and improve immune resilience, and ultimately clinical outcomes and quality of life for this vulnerable population.

## 1 INTRODUCTION

The global population is aging rapidly, with people aged 65 and over projected to reach 1.6 billion by 2050 (Ferrucci et al., 2020). This poses challenges due to chronic diseases, cognitive and physical decline, and complex medication regimens (Khan et al., 2024). Aging involves significant variability, influenced by both intrinsic factors like genetic mutations and extrinsic factors such as diet, stress, pollution, and smoking (SanMiguel et al., 2020).

Aging weakens the immune system, leading to immunosenescence and inflammaging, which compromise the body's ability to fight infections (Zheng et al., 2020). This was evident during the COVID‐19 pandemic, where older adults experienced higher rates of severe disease and death (Özgüç et al., 2024). Chronic inflammation in aging may exacerbate COVID‐19 outcomes by triggering a cytokine storm, leading to tissue damage (Tizazu et al., 2022).

This review aims to highlight aging‐related immunological changes and their implications for COVID‐19 responses, including their influence on susceptibility to infection, severity of illness, and recovery outcomes.

Unlike many existing reviews, it delves deeper into how these alterations affect clinical outcomes in older populations. Notably, this manuscript emphasizes findings from recent human‐based studies, offering a targeted perspective on the implications of aging for COVID‐19 responses.

## 2 METHODS

### 2.1 Literature search strategy

A literature search was conducted using scientific databases with MeSH‐compliant keywords on aging, immune system, and COVID‐19. Data extraction analyzed immune cell function, inflammatory markers, and clinical outcomes in the elderly. Detailed methodology is in Appendix S1.

Tables S1 and S2 summarize key findings about the effects of aging on innate and adaptive immunity, respectively (Appendix S1).

## 3 IMPACT OF AGING ON THE IMMUNE SYSTEM

Aging weakens the immune system, reducing its ability to defend against infections (Figure 3). It is the main factor behind immune deficiency (Brauning et al., 2022; Haynes, 2020).

**FIGURE 1:**
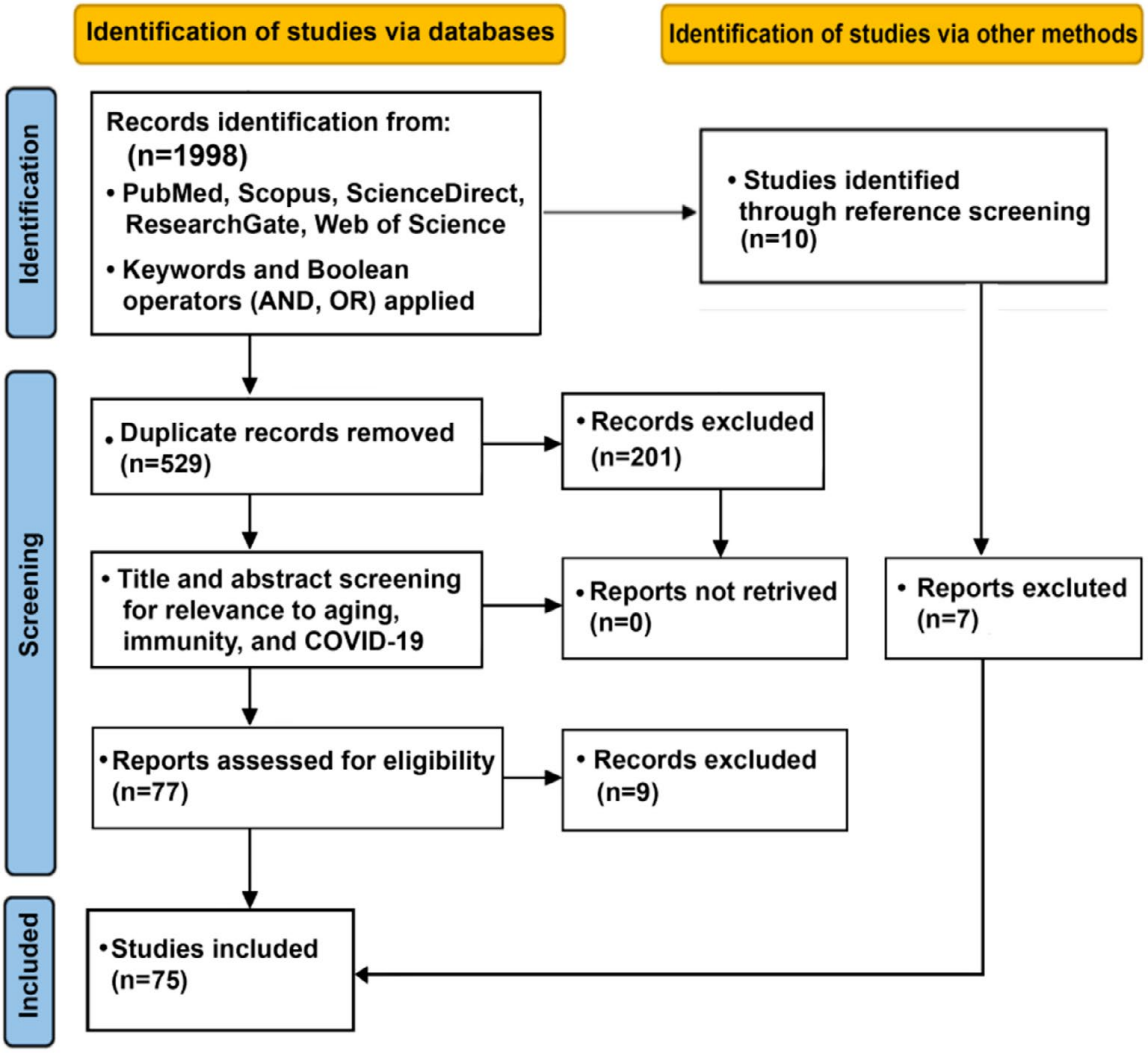
PRISMA 2020 flow diagram for the systematic review including the identification process, screening, and final number of studies included.

### 3.1 Aging of innate immune defense system

The innate immune system is the first line of defense, offering rapid, nonspecific protection through physical and chemical barriers, such as the skin, mucous membranes, and immune cells like macrophages, neutrophils, and natural killer cells (Müller et al., 2019).

#### 3.1.1 Physical barriers: Skin and mucous barriers

Skin and mucosal barriers prevent microbial entry (Berni Canani et al., 2024). With aging, skin loses elasticity and moisture, the dermis and epidermis thin, and sweat glands and blood vessels decline, increasing infection risk (Jiao et al., 2024; Park, 2022). Chronic kidney disease (CKD) and dry skin further impair the skin barrier (Gagnon & Desai, 2013; Molina et al., 2023), as uremic toxins and electrolyte imbalances disrupt function (Lim et al., 2021; Molina et al., 2023). Inflammation, oxidative stress, and pruritus exacerbate dryness, infection risk, and delayed healing, while IL‐6 and TNF‐α hinder moisture retention and protection (Zhai et al., 2024). Aging weakens the intestinal mucosa, reducing mucus production and facilitating bacterial infiltration (Herath et al., 2020). Structural changes—longer villi, fewer Paneth cells, and increased colonic crypt apoptosis—further compromise the barrier (Funk et al., 2020). Microbiota alterations lead to dysbiosis, chronic inflammation, and permeability, allowing pro‐inflammatory cytokines, bacteria, and toxins into circulation (Di Vincenzo et al., 2024). In the respiratory tract, reduced ciliated cell function and increased mucus viscosity impair pathogen clearance, heightening infection risk (Bailey, 2022; Ho et al., 2001). In postmenopausal women, estrogen decline thins the vaginal epithelium, increasing susceptibility to infection (Davis et al., 2023).

#### 3.1.2 Complement system

The complement system plays a dual role in aging, balancing protective and detrimental effects through inflammation, metabolism, apoptosis, mitochondrial function, and Wnt signaling (Zheng et al., 2022). Age‐related inflammation activates the complement system via C3 in the alternative pathway (Cao et al., 2020). Metabolic changes and caloric accumulation raise C3, contributing to obesity, diabetes, and cardiovascular disease (Engstrom et al., 2005; Jura & Kozak, 2016; Onat et al., 2011). Excessive complement activation worsens arterial plaques and coronary events (Wunder, 1992). The complement system enhances Wnt/β‐catenin signaling via C1q, contributing to muscle atrophy, renal senescence, inflammation, and fibrosis (Castellano et al., 2019). C3a promotes ROS production and NLRP3 inflammasome activation, driving chronic inflammation (Sho & Xu, 2019; Asgari et al., 2013). TNF‐α, IL‐1β and protein synthesis in peripheral blood mononuclear cells are influenced by C3a, reinforcing its proinflammatory role (Takabayashi et al., 1996).

#### 3.1.3 Inflammation

Aged monocytes adopt a pro‐inflammatory state (Hearps et al., 2012; Sadeghi et al., 1999), and overall, aging is characterized by chronic low‐grade inflammation, or inflammaging, marked by elevated levels of IL‐6, IL‐1β, TNF‐α, and CRP, increasing frailty and disease risk (Bernardi et al., 2020; Tylutka et al., 2024). Aging also increases oxidative stress (Gorni & Finco, 2020) and intestinal permeability, fueling systemic inflammation (Quin et al., 2024). Impaired apoptotic cell clearance prolongs inflammation and tissue damage (Devitt & Marshall, 2011; Larbi et al., 2005), while senescent cells secrete SASP factors, worsening inflammation (Wajapeyee et al., 2008). NLRP3 inflammasome hyperactivation promotes chronic inflammation through IL‐1β and IL‐18, leading to pyroptosis and tissue damage (Coombs et al., 2024; Savage et al., 2012; Shi et al., 2015). Metabolic changes, such as increased visceral adipose tissue and poor diet, exacerbate inflammaging and weaken immune responses, including vaccine efficacy (De Sanctis et al., 2025; Teissier et al., 2022). Chronic inflammation contributes to cardiovascular disease, neurodegeneration, diabetes, and cancer (Franceschi et al., 2000; Krabbe et al., 2004). Immune dysfunction, such as reduced macrophage clearance and naïve T‐cell depletion, worsens surveillance (Sagiv & Krizhanovsky, 2013; van Deursen, 2014). Inflammaging and cellular senescence create a feedback loop, impairing immune surveillance and autophagy, thus accelerating immunosenescence (Franceschi et al., 2000).

#### 3.1.4 Mast cells

Mast cells (MCs) play a role in angiogenesis, pathogen clearance, and vasodilation. Upon activation, they release proinflammatory peptidases and cytokines, initiating immune responses. Aging increases MCs by 40% in the papillary dermis of individuals ≥75 years compared to those ≤30 years (Kundu et al., 2020; Pilkington et al., 2019). MC degranulation is lower in older skin, likely due to reduced tachykinin precursor 1 (TAC1) gene expression, which encodes substance P, an MC activator (Pilkington et al., 2019). In older skin, MCs interact more with macrophages and nerve fibers than in young skin (Pilkington et al., 2019). MC activity contributes to the persistence of inflammaging. In postmenopausal women, the decline in estrogen levels further enhances mast cell activity, raising susceptibility to inflammation and pain (Franceschi et al., 2017).

#### 3.1.5 Dendritic cells

Dendritic cells (DCs) detect pathogens, activate naive T cells, and regulate B and NK cell responses (Sadeghi et al., 2024). They are classified into plasmacytoid DCs (pDCs) and myeloid DCs (mDCs) (Mellman, 2013). In elderly individuals, pDCs decrease due to reduced lymphoid cell hematopoiesis, while mDCs remain stable (Agrawal et al., 2007; Jing et al., 2009; Metcalf et al., 2015; Pérez‐Cabezas et al., 2007). Aging impairs DC migration, antigen phagocytosis, and cytokine production (Cui et al., 2024; Macal et al., 2018). Monocyte‐derived DCs (MoDCs) show increased pro‐inflammatory cytokines (TNF‐α, IL‐6, CXCL‐10) and reduced IL‐10 production with age (Agrawal et al., 2007). Aged MoDCs also have impaired type I/III interferon production, reducing antiviral responses (Prakash et al., 2013). These changes cause chronic inflammation and reduce immune tolerance, weakening defenses against infections such as influenza, pneumonia, herpes zoster, tuberculosis, and COVID (Agrawal et al., 2017; Agrawal & Gupta, 2011; Levin, 2012; Aravindhan & Yuvaraj, 2024; Oh & Hurt, 2014; Mueller et al., 2020; Chandra et al., 2022).

#### 3.1.6 NK cells

Natural Killer (NK) cells eliminate tumor and infected cells (Huntington et al., 2020). They secrete IFN‐γ, TNF‐α, GM‐CSF, and IL‐10, influencing the adaptive immune system (Pierce et al., 2020). NK cells are divided into two subpopulations: CD56dimCD16+ NK cells (cytotoxic with low cytokine production) and CD56brightCD16–NK cells (high cytokine production, found in tissues like bone marrow and liver) (Sun et al., 2011). In individuals over 60, NK cell numbers rise but are accompanied by altered CD69 expression, impaired cytotoxicity, and reduced cytokine production, resulting in unchanged overall cytotoxicity (Borrego et al., 1999; Chidrawar et al., 2006; Muzzioli et al., 2009; Ogata et al., 1997). Moreover, there is a decline in CD56bright NK cells, crucial for cytokine production (Almeida‐Oliveira et al., 2011; Brauning et al., 2022; Solana et al., 2014). The effect of aging on adhesion or chemokine receptors is debated, with some studies reporting a decline (Almeida‐Oliveira et al., 2011) and others no change (Le Garff‐Tavernier et al., 2010). Thus, while NK cell numbers increase, their function declines, heightening the risk of infections and cancer (Le Garff‐Tavernier et al., 2010).

#### 3.1.7 Neutrophils

Neutrophils, key phagocytic cells in infection control and injury healing, are attracted to infection sites by cytokines and chemokines. Although their numbers remain constant with age, in the elderly, their chemotactic and phagocytic abilities are reduced (Aroca‐Crevillén et al., 2024; Butcher et al., 2001; Qian et al., 2014; Wenisch et al., 2000), along with a diminished bactericidal capacity of neutrophil extracellular traps (NETs) (Hazeldine et al., 2014; Sabbatini et al., 2022). The decline in their function is due in part to a reduced expression of CD16 (FC receptor), which is essential for the phagocytosis of opsonized bacteria and the generation of superoxide (Butcher et al., 2001). Although key in age‐related diseases (Van Avondt et al., 2023), neutrophil decline is not directly linked to them. Understanding their role is crucial for developing targeted therapies for older adults.

#### 3.1.8 Eosinophils and basophils

Compared to the extensive data on neutrophils, the effect of aging on eosinophils and basophils has been much less studied (Annema et al., 1995; Marone et al., 1986; Mathur et al., 2008; Schwarzenbach et al., 1982; Sokol et al., 2009; Uciechowski & Rink, 2014). Some of the functions of eosinophils and basophils impaired with age are presented in Figure 1.

**FIGURE 2:**
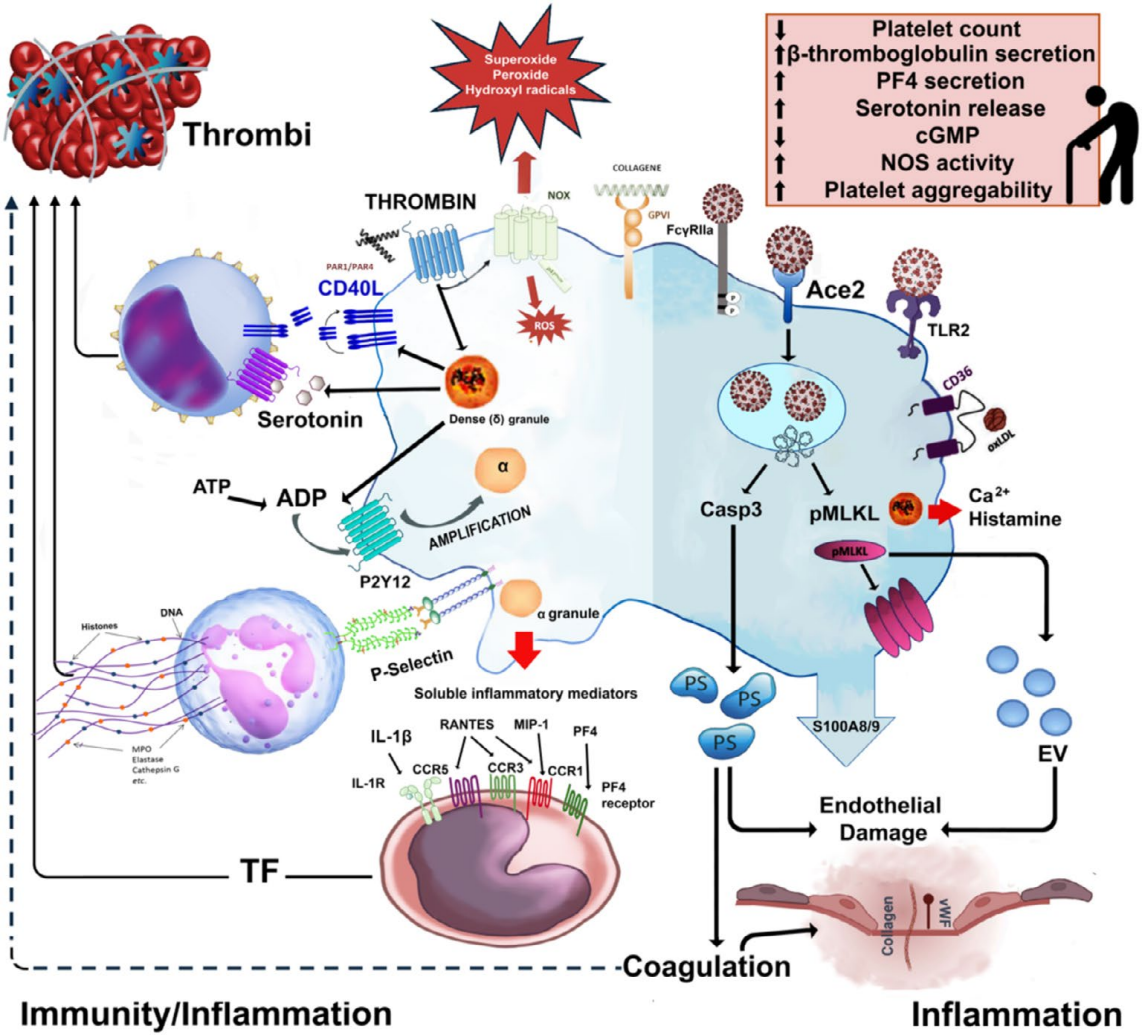
Primary alterations in platelet function and behavior associated with aging during COVID‐19 infection.

#### 3.1.9 Platelet

Platelets internalize microbes, generate antimicrobial ROS, and mobilize granules via microtubule assembly, similar to neutrophils and macrophages (Yeaman, 2010). Platelet count declines with age (Segal & Moliterno, 2006), and lower counts are linked to higher mortality in older adults (Le Blanc & Lordkipanidzé, 2019). Aging increases platelet hyperactivity, with heightened aggregation sensitivity to ADP (Bastyr 3rd et al., 1990; O'Donnell et al., 2001), epinephrine (Kasjanovová et al., 1993), collagen (Kasjanovová & Baláz, 1986), and arachidonic acid (Kasjanovová et al., 1993). Women show higher aggregability at all ages (Kasjanovová et al., 1993; Meade et al., 1985), and age‐related changes in platelet behavior on von Willebrand factor are more pronounced in women (Cowman et al., 2015).

In the elderly, hyperaggregability is associated with increased β‐thromboglobulin and PF4 secretion from α‐granules (Bastyr 3rd et al., 1990; Zahavi et al., 1980), potentially enhancing serotonin release and contributing to atherothrombotic disease (Gleerup & Winther, 1988). ROS act as second messengers or generate oxidized proteins, contributing to platelet activation and potentially increasing thrombotic risk in aging (Fuentes et al., 2017; Jang et al., 2014). Disruption of integrin αIIbβ3 activation may further alter thrombotic susceptibility (Levin et al., 2013). Oxidative stress promotes thrombus formation via various mechanisms (Butera et al., 2014; Fuentes & Palomo, 2016; Violi et al., 2017). NO inhibits platelet aggregation through GC activation and cGMP production, reducing intracellular calcium (Fuentes & Palomo, 2016), but it can also generate ONOO–, leading to oxidative stress and cellular damage (Bartesaghi & Radi, 2018). Aging is linked to reduced platelet cGMP levels despite increased NOS activity, possibly due to ONOO–‐mediated GC inhibition (Kawamoto et al., 2005). The platelet transcriptome adapts to disease states (Best et al., 2017), with RNA sequencing revealing 514 differentially expressed transcripts in young (<45 years) versus elderly (>64 years) individuals (Campbell et al., 2018). Age‐related platelet proteome changes remain unclear (Cini et al., 2015), indicating complex transcriptome regulation in aging. Figure 2 summarizes the key changes in platelet function and behavior that occur with aging.

**FIGURE 3:**
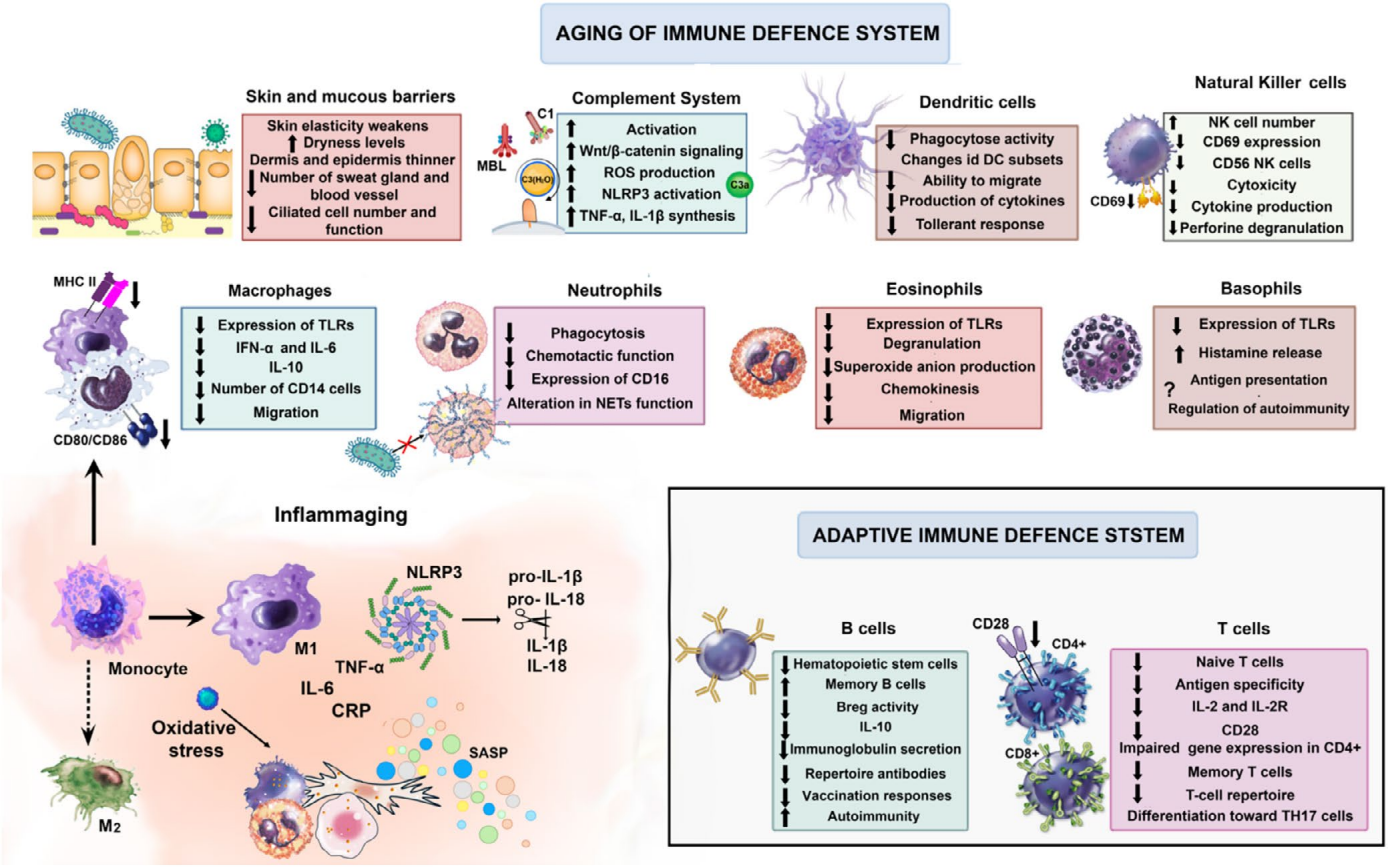
Aging of immune defense system.

#### 3.1.10 Macrophages

Macrophages express toll‐like receptors (TLRs) to recognize antigens and attract neutrophils by producing chemokines and cytokines (IL‐1, IL‐6, IL‐8, TNF‐alpha). They also present antigens to T lymphocytes, regulating the adaptive immune response (Denes, 2024; Joshi et al., 2018). In aged macrophages, impaired antigen presentation (with reduced CD80, CD86, and MHC‐II) and lower superoxide anion production after IFN‐γ treatment diminish antimicrobial activity (Franceschi et al., 2000; Solana et al., 2012; Stout & Suttles, 2005). Additionally, increased CD14 and TLR expression in older individuals suggests impaired phagocytosis and reduced reactive oxygen species generation (van Beek et al., 2019; De Maeyer & Chambers, 2021). Macrophage senescence contributes to immune dysfunction and age‐related diseases (infections, autoimmune disorders, cancers (Wang et al., 2024), arthritis, and cardiovascular diseases). Understanding these alterations may pave the way for new drugs targeting senescent macrophages to improve well‐being in aging.

### 3.2 Aging of adaptive immune defense system

Adaptive immunity specifically recognizes microorganisms and foreign substances through antigen receptors on B lymphocytes and T lymphocytes.

#### 3.2.1 T lymphocytes

T lymphocytes originate from hematopoietic stem cells in bone marrow and mature in the thymus. They are divided into CD4+ (T‐helper) and CD8+ (cytotoxic) subsets. CD4 + T cells recognize antigens via MHC II, while CD8 + T cells bind MHC I antigens to target malignant or infected cells (Fabbri et al., 2003; MacIver et al., 2013). With advancing age, there is a notable decline in T cell number, linked to thymic involution, which leads to reduced production of new T cells (Fujimori & Ohigashi, 2024; Palmer et al., 2018). T cells undergo significant changes, including reduced antigen specificity and TCR expression, leading to impaired TCR‐triggered gene expression in CD4+ T cells (Bektas et al., 2013; Chen et al., 2013; Naylor et al., 2005). In addition, the expression of co‐stimulatory CD28 decreases with age due to changes in its gene promoter (Chiu et al., 2006; Merino et al., 1998). IL‐2 and IL‐2R expression consistently decreases with age (Gillis et al., 1981). However, the dysregulation of the Th1 cytokine IFN‐γ and the Th2 cytokines IL‐4 and IL‐5 in aged humans is not as clear. Some reports showed that these cytokines were increased in aged humans, while others showed decreased cytokine expression by aged human T cells (Bandrés et al., 2000; Karanfilov et al., 1999). Aging T cell differentiation biases toward TH17 cells, which reshapes immunity in elderly individuals (Ouyang et al., 2011).

With aging, the adaptive immune response weakens as the T‐cell repertoire becomes less diverse. Naive T cells mature into memory effector cells, particularly CD8+ cells, acquiring markers like CD56 and increasing cytotoxic potential. CD28—T cells can be activated without TCR signaling, but their proliferation is reduced, diminishing immune function in older individuals (Chiu et al., 2006; Guan et al., 2024; Merino et al., 1998). Aging also reduces mitochondrial function, lowering ATP production and increasing oxidative stress, which induces mesenchymal senescence (Brandl et al., 2011), compromises T‐cell activation and differentiation (Boyd et al., 2012; Lane et al., 2015), and facilitates tumor progression with poor clinical outcomes in older individuals (Han et al., 2023).

#### 3.2.2 B lymphocytes

Aging decreases naive, pro‐, pre‐, and immature B cells (Cancro et al., 2009) due to intrinsic B cell aging (Guerrettaz et al., 2008; Stephan et al., 1997) and reduced hematopoietic stem cell potential (Cancro, 2020; Frasca et al., 2020). Although memory B cells persist, their differentiation is impaired in older adults due to low Blimp‐1 expression (Frasca et al., 2016). An atypical, non‐dividing subset—age‐associated B cells—accumulates with age; these cells present antigens, secrete cytokines and antibodies, and respond to innate receptor stimulation but are unresponsive to BCR stimulation (Hao et al., 2011). Some studies report an increase in memory B cells (CD27+) due to somatic mutations and resistance to apoptosis (Ciocca et al., 2021). Regulatory B cells (Breg) produce IL‐10, IL‐35, or TGF‐β (Glass et al., 2022), yet reduced Breg activity in aging may increase chronic inflammation and lower IL‐10 (Knippenberg et al., 2011). Plasma cell numbers decline with age, reducing immunoglobulin secretion (Frasca et al., 2011); while plasma cells still secrete antibodies, their effectiveness is diminished—leading to slower responses after vaccination and higher infection susceptibility (Andreu‐Sánchez et al., 2024; Clark et al., 2012; Frasca & Blomberg, 2020; Gustafson et al., 2020). Somatic hypermutation remains active, but opsonin production for bacterial neutralization is reduced (Yu et al., 2024), resulting in decreased antibody responses to novel pathogens and increased autoimmunity (Teissier et al., 2022).

## 4 IMMUNITY RESPONSE TO COVID‐19 IN ELDERLY PEOPLE

### 4.1 Innate response to COVID‐19 in elderly people

#### 4.1.1 Skin and mucosal barriers

Aging reduces the number and function of ciliated respiratory cells, impairing mucociliary clearance and increasing lung infection risk (Bailey, 2022; Ho et al., 2001). In elderly COVID‐19 patients, decreased mucus clearance and increased secretion viscosity promote pathogen accumulation and lung inflammation (Adivitiya et al., 2021). SARS‐CoV‐2 infects cells via the ACE2 receptor, which is abundant in alveolar type 2 and bronchial ciliated cells, making these tissues major infection targets (Morrison et al., 2022). Severe COVID‐19 cases often show a higher IL‐6/interferon‐γ ratio, contributing to a cytokine storm and lung injury (Wang et al., 2020), while elevated IL‐6 levels, linked to age‐related frailty and higher mortality, worsen disease severity (Avila‐Nava et al., 2021; Guirao et al., 2020). The air‐blood barrier's integrity is crucial for lung health (Johnston et al., 2021), yet SARS‐CoV‐2 can damage the airway epithelium and trigger a harmful inflammatory cycle, especially in older people (Josset et al., 2013; Xu et al., 2024).

#### 4.1.2 Inflammation

Type I interferons (IFN‐I) produced by various cells counter viral spread (Sacchi et al., 2023). However, aging impairs RIG‐I signaling, reducing IFN gene expression (Molony et al., 2017) as seen in SARS‐CoV‐1 studies (Pietrobon et al., 2020). Older adults have fewer pDCs with lower TLR7 expression and impaired IFN‐I production (Greene et al., 2025), weakening defenses against influenza A and COVID‐19 (Pietrobon et al., 2020).

COVID‐19 severity is linked to ACE2, the receptor for SARS‐CoV‐2 (Elnagdy et al., 2024). ACE2 converts pro‐inflammatory Ang II into anti‐inflammatory Ang 1–7, lowering IL‐6, TNF‐α, and IL‐8 while raising IL‐10 (Klein et al., 2013). However, elevated Ang II in severe cases contributes to cytokine storms (Gheblawi et al., 2020). Older adults and patients with cardiovascular disease or diabetes have lower ACE2 levels, which may reduce viral entry but lead to an exaggerated pro‐inflammatory response that worsens acute lung injury and ARDS (Tikellis & Thomas, 2012). Elevated Ang II in severely ill COVID‐19 patients supports this hypothesis (Valle Martins et al., 2021).

#### 4.1.3 Complement system

Aging also dysregulates complement activation, worsening COVID‐19 outcomes (Noris et al., 2020) and increasing the risk of cytokine storms and multiorgan damage (Meroni et al., 2023). Uncontrolled activation of C3 and C5 exacerbates tissue damage and systemic inflammation. High plasma levels of sC5b‐9, C3a, and factor Bb, along with low MBL, are linked to higher mortality. The MBL pathway is crucial for early antiviral response, but age‐related declines in MBL and IgM reduce immune protection and heighten SARS‐CoV‐2 susceptibility (Boechat et al., 2021; Muthana & Gildersleeve, 2016). Therapies that restore MBL levels or modulate complement activation (e.g., C3 and C5 inhibitors) could enhance immune responses in older patients.

#### 4.1.4 Mast cell

SARS‐CoV‐2 can activate mast cells (MCs), triggering the release of inflammatory mediators (Conti et al., 2020). In older adults, dysregulated MC activation leads to excessive production of histamine, tryptase, chymase, prostaglandins, leukotrienes, IL‐6, and TNF‐α, contributing to cytokine storms, chronic inflammation (Afrin et al., 2020; Kritas et al., 2020), endothelial damage, and ARDS (Dileepan et al., 2023; Galli et al., 2020). Excessive MC activation exacerbates lung inflammation, worsening respiratory failure (Theoharides, 2021), and is linked to more severe COVID‐19 outcomes (De Maeyer et al., 2020). It also increases vascular permeability and thrombosis, common complications in severe COVID‐19 (Barnes et al., 2019). Targeting MC‐associated pathways, such as IL‐6/JAK–STAT signaling, or using MC stabilizers could help control excessive inflammation in elderly COVID‐19 patients (Hafezi et al., 2021).

#### 4.1.5 Dendritic cell

When stimulated ex vivo, DCs taken from the blood of patients with COVID‐19 had minimal expression of CD80, CD86, C–C motif chemokine receptor (CCR)7 and human leukocyte antigen (HLA)‐DR (Zhou et al., 2020), compromising their ability to stimulate adaptive immunity (Wong & Goldstein, 2013). Infections, such as SARS‐CoV‐1, can activate DCs to produce inflammatory cytokines (TNF‐α, IL‐6) and chemokines (MIP‐1α, RANTES, IP‐10, MCP‐1, CCL3, CXCL10) (Agrawal et al., 2007; Law et al., 2005). In the elderly, viral escape mechanisms inhibit the production of antiviral cytokines (IFN‐α, IFN‐β) (Law et al., 2005; Prakash et al., 2013; Zhou et al., 2020) due to reduced total numbers of pDCs and their defective TLR7/TLR9 signaling (Jing et al., 2009). In SARS‐CoV‐2 infection, impaired IFN‐I responses correlate with disease severity (Hadjadj et al., 2020).

#### 4.1.6 NK cell

With aging, NK cell cytotoxicity decreases, also because they express higher levels of the inhibitory receptor NKG2A, reducing the ability to eliminate virus‐infected cells (Zheng et al., 2020). In COVID‐19, NK cell function is impaired due to the delayed production of IFN‐I, which occurs in elderly individuals, and to their reduced responsiveness to IFN‐I, further compromising their antiviral function (Acharya et al., 2020; Shaw et al., 2013). In addition, NK cells from elderly individuals have a lower expression of perforin and granzyme B, which are necessary for their cytotoxic activity. Impaired NK cell function in elderly individuals is associated with lower infection rates and reduced chances of resolving the infection at an earlier stage compared to younger individuals (Acharya et al., 2020; Wilk et al., 2020).

#### 4.1.7 Neutrophils

In elderly COVID‐19 patients, increased peripheral blood neutrophils correlate with disease severity and worse prognosis (Ince et al., 2024). Elevated IL‐6 levels, caused by COVID‐19 or age‐related inflammation, prolong neutrophil survival by inhibiting apoptosis through altered JAK–STAT and PI3K‐AKT signaling. Older neutrophils exhibit hyperactivation of the PI3K pathway, leading to misdirected migration and spread into uninfected tissues, contributing to chronic inflammation (Larbi et al., 2005). In COVID‐19, older neutrophils show excessive degranulation, promoting cytokine storms, endothelial damage, and thrombosis, worsening severity (Gullotta et al., 2023). Excessive neutrophil activation leads to lung injury, increased vascular permeability, and ARDS. Dysregulated neutrophil function in aging, combined with a pro‐inflammatory environment, results in poorer outcomes and higher mortality in elderly COVID‐19 patients.

#### 4.1.8 Eosinophils and basophils

Eosinophils and basophils play crucial roles in the immune response to COVID‐19. In older adults, low eosinophil levels are associated with reduced antiviral effects and severe COVID‐19 (Gambichler et al., 2023). Basophil depletion, particularly during the acute phase of COVID‐19, reduces humoral memory activation (Murdaca et al., 2021). Eosinopenia and basophilia are reliable biomarkers for predicting severe and fatal outcomes in COVID‐19, with reduced granulocyte levels linked to a worse prognosis (Cazzaniga et al., 2021; Ito et al., 2023).

#### 4.1.9 Macrophages

Upon virus detection, macrophages release pro‐inflammatory cytokines that inhibit viral replication, activate adaptive immunity, and recruit immune cells (Kosyreva et al., 2021). Increased monocyte apoptosis in elderly COVID‐19 patients contributes to lymphopenia and weak immune responses (Abiri et al., 2024), partly due to reduced monocyte TLR expression (Onofrio et al., 2020; Shaw et al., 2013). Aging impairs macrophage function, with lower MHC class II expression after IFN‐γ stimulation in older individuals. COVID‐19 disrupts the M1/M2 balance, as CD14 + CD16+ macrophages produce high levels of inflammatory cytokines (TNF‐α, IL‐6) and chemokines (CCL2, CCL3, CCL5, CXCL10) (Acharya et al., 2020; Hearps et al., 2012; Wong et al., 2011). In elderly lungs, monocytes/macrophages secrete more IL‐6 but fail to produce antiviral cytokines (IFN‐α, IFN‐β, IFN‐γ, IL‐12p40), impairing CD8+ cell activation and facilitating immune evasion (Velazquez‐Salinas et al., 2019). Impaired interferon responses worsen COVID‐19 outcomes, partly due to a lack of airway repair macrophages (Hadjadj et al., 2020; Aiello et al., 2019; Acharya et al., 2020). Aging also reduces alveolar macrophage populations and enhances inflammatory responses, as shown by increased proinflammatory cytokine secretion in response to Mycobacterium tuberculosis and resistance to IFN‐γ stimulation (Hussell & Bell, 2014; McQuattie‐Pimentel et al., 2021).

#### 4.1.10 Platelets

Platelets are involved in a variety of mechanisms during SARS‐CoV‐2 infection. Platelet activation and thrombus formation can worsen thrombosis, while viral activation and cell death pathways amplify inflammation, leading to complications like thrombosis and organ damage (Rondina et al., 2013). In COVID‐19 patients, platelets are more prone to aggregation in response to stimuli like ADP, epinephrine, and other molecules (Lê et al., 2015; Zaid et al., 2020). However, despite this heightened reactivity, platelets show a reduction in granule contents, such as PF4 and serotonin, suggesting prior activation (Zaid et al., 2020). Molecules like sCD40LG, TxB2, and vWF in plasma indicate platelet activation, with markers such as P‐selectin and CD40 elevated in COVID‐19 patients (Hottz et al., 2020; Nunez‐Avellaneda et al., 2018; Zaid et al., 2020). Platelets form Heterotypic Aggregates (HAGs) with leukocytes, promoting tissue factor (TF) expression, a key coagulation activator. Platelets interact with CD4 and CD8 T cells, but the role of these interactions in immune modulation is unclear and needs further study (Scherlinger et al., 2023). Platelets contribute to “immunothrombosis” in COVID‐19 by surrounding NETs, leading to microthrombi in organs like the lungs, kidneys, and heart, worsening thrombotic complications (Colicchia et al., 2022; Conway et al., 2022). SARS‐CoV‐2 vRNA in platelets is linked to severe disease. While the virus enters via ACE2, it can also enter through alternative routes, such as viral particles on microparticles or via the FcγRIIa receptor (Lim et al., 2022). This may explain vRNA persistence for up to 19 days (Gaspar‐Rodríguez et al., 2021). Platelets have TLRs that recognize SARS‐CoV‐2. TLR2 can be activated by the virus, increasing platelet aggregation and HAG formation. Upon virus exposure, platelets activate apoptosis (via Casp3) and necroptosis (via pMLKL), causing membrane rupture and the release of microparticles and exosomes, which promote aggregate formation and inflammatory cytokine release (Sciaudone et al., 2023). After necroptosis and pyroptosis, platelets release DAMPs like calprotectin (S100A8/A9), which drive inflammation and endothelial activation, leading to vessel damage and thrombus formation. S100A8/A9 also promotes platelet microvesicle generation, enhancing coagulation and cytokine release.

### 4.2 Adaptive response to COVID‐19 in elderly people

#### 4.2.1 Cellular immune response to COVID‐19 in elderly people

The adaptive immune response in COVID‐19 relies on cellular immunity. Young patients show a significant increase in activated CD8+ T cells between days 7 and 9 post‐infection, coinciding with symptom resolution (Thevarajan et al., 2020). In contrast, elderly patients experience marked decreases in CD4+ and CD8+ T cells, correlating with lower survival rates (Diao et al., 2020; Westmeier et al., 2020), and T cell numbers are negatively correlated with IL‐6, IL‐10, and TNF‐α levels (Diao et al., 2020), with elevated IL‐6 reducing CD4+ T cells and NK cells. Older adults also show fewer naive T cells, impairing their SARS‐CoV‐2 response and leading to more severe outcomes (Nicoli et al., 2020; Schwartz et al., 2020). Thymic involution reduces T cell production and TCR diversity (Fujimori & Ohigashi, 2024), and studies link reduced TCR diversity with severe COVID‐19, as older individuals struggle to generate effective naive T cells (Goronzy et al., 2015). Additionally, aging decreases Treg cells, crucial for regulating *cytokine storms*, further exacerbating inflammation and severe disease outcomes.

#### 4.2.2 Humoral immune response to COVID‐19 in elderly people

Aging reduces the effectiveness of the humoral immune response due to changes in B cells, which lose their capacity for somatic hypermutation (Frasca et al., 2011). This impairs older adults' ability to produce high levels of neutralizing antibodies, weakening immunity against infections (Weiskopf et al., 2009; Weksler et al., 2002). Aging also affects the production of class‐switched antibodies necessary for combating viral infections and leads to the accumulation of aged B cells with unique properties (Carey et al., 2024). Additionally, key signaling molecules like CD40L decline with age, hindering T cell–B cell interactions (Gruver et al., 2007). Older adults, similar to individuals with severe COVID‐19, often experience lymphopenia, reducing T cell numbers (Michels et al., 2024), further compromising immune responses. As a result, they struggle to mount a strong defense against infections like COVID‐19, which leads to more severe disease outcomes.

The comparison between the immune system in young and elderly individuals and the relationship between aging and inflammatory factors in COVID‐19 is detailed in Table S3 (Appendix S1).

**FIGURE 4:**
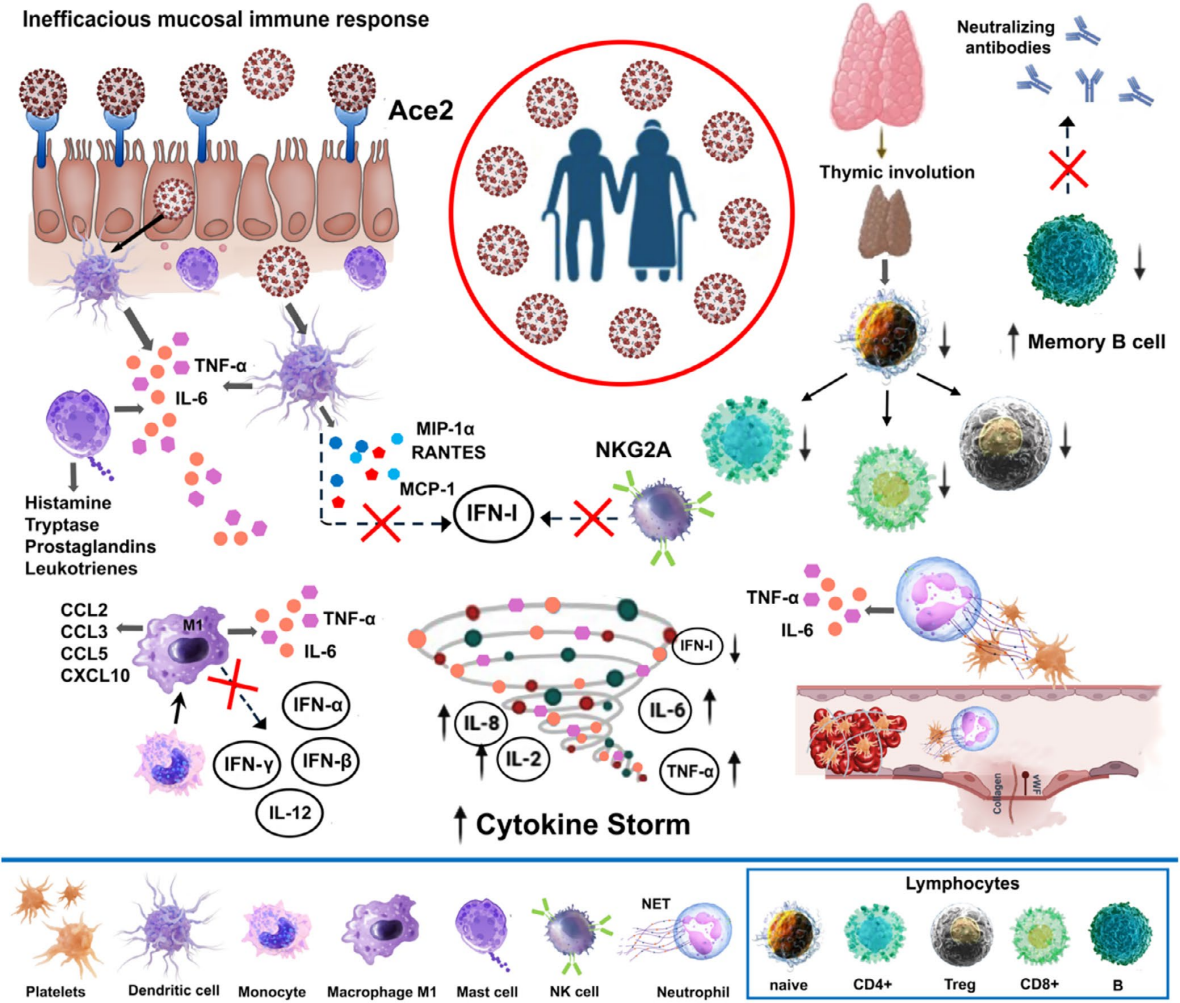
COVID‐19 Immune Response in elderly individuals.

Finally, Figure 4 shows a summary of the key events in the immune response to COVID‐19 in the elderly.

## 5 CONCLUSION

The review underscores the role of age‐related immune changes in COVID‐19 outcomes. A key limitation is the lack of large‐scale longitudinal studies of COVID‐19 immunity in older adults, which limits generalizability. While immune aging varies among individuals, no studies have specifically addressed this in COVID‐19. Future research should explore its impact on susceptibility, severity, and recovery. Promising interventions include lifestyle changes and physical activity, but further research is needed. Future studies should focus on optimizing vaccines and therapies targeting inflammation, mitochondrial dysfunction, and oxidative stress to enhance immune resilience in older adults, particularly those with comorbidities.

## AUTHOR CONTRIBUTIONS

M.G.: Conceived and designed research, Prepared figures, Drafted manuscript, Edited and revised manuscript; M.H.: Drafted manuscript, Edited and revised manuscript; A. M.: Prepared figures, Drafted manuscript, Edited and revised manuscript; S.M.: Drafted manuscript, Edited and revised manuscript; A.S.: Conceived and designed research, Drafted manuscript, Edited and revised manuscript. All authors approved the final version of themanuscript.

## FUNDING INFORMATION

This research received no external funding.

## CONFLICT OF INTEREST STATEMENT

No conflicts of interest, financial or otherwise, are declared by the authors.

## ETHICS STATEMENT

This article is a review of previously published studies and does not involve any original data collection involving human or animal subjects. Therefore, ethical approval and informed consent were not required.

## Supporting information


Appendix S1.
