# Supplementary material for: Aging‐associated changes in immunological parameters: Implications for COVID‐19 immune response in the elderly
Source: Physiol Rep. 2025 May 22;13(10):e70364. doi: 10.14814/phy2.70364 (PMC12098970; doi:10.14814/phy2.70364)
Supplement: Supplementary file 1 — Appendix S1. [file PHY2-13-e70364-s001.docx]

**2. Methods**

**2.1 Literature Search Strategy**

For this narrative review, a literature search was performed using scientific articles, bibliographic indexes, and electronic databases, including PubMed, Scopus, CINAHL, Embase, ScienceDirect, ResearchGate, and the Web of Science. The search aimed to identify relevant articles published up to 2024. We applied MeSH-compliant keywords relating to Aging (“Aged”, “Aging Physiology”, “Geriatrics”, “Geriatric Assessment”, “Longevity”, “Biological Aging”, “Health of the Elderly”, “Healthy Aging”, “Age-Related Changes”, “Functional Decline”,  “Physical Frailty”, “Chronic Disease and Aging”, “Aging Immunity”), Immune System (“Immune System”, “Immunity”, “Immune Response”, “Immune System Diseases”, “Autoimmunity”, “Innate Immunity”, “Adaptive Immunity”, “Humoral Immunity”, “Cell-Mediated Immunity”, “Immunoglobulins”, “Cytokines”, “T-Lymphocytes (T-Cells)”, “B-Lymphocytes (B-Cells)”, “Dendritic Cells”, “Macrophages”, “Natural Killer Cells”, “Leukocytes”, “Antigen-Presenting Cells”, “Hypersensitivity”, “Immunotherapy”, “Vaccines”, “Inflammation”), COVID-19 ("Here are MeSH-compliant keywords related to COVID-19 (“COVID-19”, “COVID-19 Vaccines”, “SARS-CoV-2”, “Coronavirus Infections”, “Pandemics”, “Severe Acute Respiratory Syndrome Coronavirus 2 (SARS-CoV-2”, “Respiratory Tract Infections”, “Antibodies, Viral”, “Antiviral Agents”, ”Vaccination”,”Immunization Programs”, “Disease Transmission, Infectious”, “Post-Acute COVID-19 Syndrome”). The use of Boolean operators (AND, OR) facilitated the effective combination of these keywords. Furthermore, a manual review of the reference lists of pertinent articles was conducted to identify additional studies aligning with the review's inclusion criteria.

**2.2 Inclusion and Exclusion Criteria**

Studies integrated into this narrative review adhered to the following criteria: direct relevance to aging and its impact on the immune system, hematological parameters, and the aging-associated alterations on hematological and immune responses to COVID-19 in elderly people; and accessibility of full-text articles in English. On the contrary, exclusion criteria encompassed studies with insufficient or unrelated content, publications not available in the English language.

This stringent selection process aimed to ensure that the included studies were not only current but also directly related to the diverse facets of aging under investigation. The use of English-language sources facilitated the comprehensive understanding and synthesis of research findings within the chosen domains.

**2.3 Data Extraction and Synthesis**

In this review, data extraction will follow a structured approach to capture the essential characteristics of studies exploring the impact of aging on immunological parameters and responses to COVID-19. Study characteristics, including author names, publication year, study design, sample size, and demographic details of elderly populations, will be documented to provide context. Immunological parameters of interest, such as cytokine levels (e.g., IL-6, TNF-α), T-cell and B-cell functionality, immunoglobulin levels, and inflammatory markers, will be extracted to assess the effects of aging on immune cells. Changes in critical immune cell types, such as T-cells, B-cells, dendritic cells, macrophages, neutrophils, and NK cells, will be analyzed, highlighting how aging affects their functions and responses to pathogens.

Data on COVID-19 outcomes, including immune responses (antibody and T-cell responses) and clinical metrics such as disease severity, mortality, recovery rates, and vaccine efficacy in elderly versus younger populations, will be collected. Studies that offer comparative analyses between elderly and younger populations, particularly in susceptibility to infection and recovery outcomes, will be included to provide insights into age-related immune response differences. Interventions aimed at improving immune responses in elderly populations, such as immune-boosting therapies and vaccination strategies, will also be documented to evaluate their potential benefits. Furthermore, data on age-related comorbidities, such as cardiovascular disease, diabetes, and chronic inflammation (inflammaging), which may influence COVID-19 outcomes, will be considered. Data synthesis will focus on organizing findings into key themes, including immunosenescence, inflammaging, altered immune responses to SARS-CoV-2, and COVID-19 vaccine response in elderly populations. This synthesis will summarize studies on aging’s impact on immune function and its implications for COVID-19. It will provide a comprehensive narrative on how aging-related changes affect clinical outcomes and potential strategies to improve health in the elderly during pandemics.

**Table S1.** Innate Immunity and Aging: Summary of Main Findings

| Study | | | | | Objective | | | | | Methods | | Main Findings | | | | | | Limitations |
| --- | --- | --- | --- | --- | --- | --- | --- | --- | --- | --- | --- | --- | --- | --- | --- | --- | --- | --- |
| Schwarzenbach et al., 1982 | | | | | Examine the effects of aging on skin reactivity, basophil degranulation, and total serum IgE levels | | | | | Skin tests with histamine, codeine, and allergens; basophil degranulation assay; serum IgE measurement in two age groups (mean ages 23.1 and 73.9 years) | | Reduced skin reactions to histamine and codeine in older individuals; no significant decline in basophil degranulation or serum IgE levels with age | | | | | | Small sample size; only non-allergic females included, limiting generalizability |
| Marone et al., 1986 | | | | | Investigate age-related changes in human basophil releasability | | | | | Analysis of IgE- and non-IgE-mediated basophil histamine release in 63 healthy donors (ages 1–86 years) | | Histamine release in response to anti-IgE correlated with donor age (r = 0.57, p < 0.001); sensitivity to anti-IgE also increased with age (r = 0.68, p < 0.001) | | | | | | Limited to histamine release; no exploration of functional consequences on allergic or inflammatory diseases |
| Gleerup & Winther, 1988 | | | | | Examine age-related changes in platelet sensitivity to serotonin | | | | | Platelet aggregation induced by adrenaline, ADP, and serotonin in 12 young (18-27 years) and 12 elderly (72-86 years) participants; serum thromboxane B2 (TXB2), 6-keto-PGF1α, plasma adrenaline, and cyclic AMP (cAMP) levels measured | | Elderly group showed significantly increased platelet aggregation in response to adrenaline (p<0.05), ADP (p<0.02), and serotonin (p<0.01); plasma adrenaline was elevated, but cAMP levels were unchanged | | | | | | Small sample size; no mechanistic insights into why serotonin sensitivity increases with age |
| Bastyr et al., 1990 | | | | | Investigate the effects of aging on platelet activity and phosphoinositide turnover | | | | | Platelets harvested from 40 healthy, non-obese individuals (ages 22–62); studies included platelet aggregation, plasma beta-thromboglobulin (β-TBG) measurement, and phosphoinositide turnover analysis using [32P]-orthophosphate incorporation | | Positive correlation between age and ADP-induced platelet aggregation, plasma β-TBG, and phosphoinositide turnover; increased thrombin-stimulated phosphatidic acid formation with age | | | | | | Study focused only on healthy individuals without atherosclerotic disease, limiting generalizability to broader populations |
| Annema et al., 1995 | | | | | Investigate the relationship between chronic respiratory symptoms, airway responsiveness, and eosinophilia in older men | | | | | Analysis of eosinophil counts, methacholine airway responsiveness, and respiratory symptoms in 894 male participants in the Normative Aging Study (mean age 60 years, range 41-90 years). | | Eosinophilia was significantly associated with airway hyperresponsiveness (OR 1.7, CI 1.1-2.7) and chronic respiratory symptoms (OR 2.0, CI 1.4-2.7). The combination of symptoms and airway responsiveness had an even stronger association (OR 3.4, CI 2.0-5.6). No significant relationship with neutrophil counts. | | | | | | Study limited to older men; causality cannot be inferred from the association; no direct evaluation of treatment effects. |
| Sadeghi et al., 1999 | | | | | Characterize phenotypic and functional changes in circulating monocytes with aging | | | | | Flow cytometry, cytokine assays, monocyte-lymphocyte co-culture | | Elderly individuals showed an expansion of CD14dim/CD16bright monocytes, indicative of activation. Increased baseline production of IL-1β, IL-6, and IL-1 receptor antagonist, but reduced IL-1β production upon stimulation. Suggests monocytes in aging are pre-activated but show impaired response to immune challenges. | | | | | | Small sample size, lacks in-depth mechanistic studies |
| Wenisch et al., 2000 | | | | | Investigate the effect of age on human neutrophil function | | | | | Assessment of neutrophil phagocytosis, reactive oxygen intermediate production, bactericidal activity, chemotaxis in three age groups (21-36, 38-56, 62-83 years) | | Significant age-dependent reduction in phagocytosis of E. coli and S. aureus (P<0.001), decreased intracellular reactive oxygen production (P<0.001), reduced neutrophil bactericidal activity (P<0.01), and impaired chemotaxis (P=0.022) in elderly subjects. Increased intracellular calcium and reduced hexose uptake were associated with these changes. | | | | | | Cross-sectional design, limited sample size in each age group, does not address causal mechanisms. |
| Ho et al., 2001 | | | | | Examine the effect of aging on nasal mucociliary clearance and ciliary function | | | | | Mucociliary clearance tests, ciliary beat frequency measurement, ultrastructural analysis | | Aging is associated with reduced nasal mucociliary clearance, lower ciliary beat frequency, and structural abnormalities in respiratory cilia | | | | | | Small sample size, cross-sectional design, and lack of longitudinal follow-up |
| Butcher et al., 2001 | | | | | Investigate the effect of aging on neutrophil bactericidal responses | | | | | Neutrophil function assays, flow cytometry, bacterial opsonization, phagocytosis assays in young (23-35 years) and elderly (>65 years) volunteers | | Neutrophils from elderly subjects showed reduced phagocytic index (P<0.005) and lower CD16 expression (P<0.0001) compared to young donors. CD16 levels correlated with phagocytic index (r=0.83; P<0.05). Superoxide generation and opsonization of E. coli were not impaired. | | | | | | Limited sample size, cross-sectional design, does not assess functional consequences on infection outcomes. |
| Larbi et al., 2005 | | | | | Investigate the role of MAPK pathway alterations in GM-CSF modulated neutrophil apoptosis with aging | | | | | Neutrophil isolation, GM-CSF stimulation, MAPK inhibitors, apoptosis assays | | The study showed that in elderly subjects, GM-CSF-induced delay in neutrophil apoptosis was reduced, linked to alterations in the p42/p44 MAPK pathway. The p38 MAPK pathway was not involved in GM-CSF-mediated apoptosis delay but played a role in spontaneous apoptosis. GM-CSF did not modulate Bcl-2 family members in elderly neutrophils as in younger ones. | | | | | | Limited to an in vitro setting, doesn't address potential in vivo implications or broader immune responses across aging populations |
| Chidrawaret al., 2006 | | | | | Assess the impact of aging on NK cell subsets | | | | | Flow cytometry, quantification of CD56bright and CD56dim NK cells | | The absolute number of peripheral blood NK (CD56+CD3−) cells remained stable with aging. CD56dim NK cells were unchanged, but CD56bright NK cells declined by 48% with age (p = 0.0004). This decline could impair cytokine secretion and immune regulation in the elderly. | | | | | | Does not explore functional consequences of CD56bright NK cell decline on overall immune responses |
| Agrawal et al., 2007 | | | | | Examine functional changes in dendritic cells (DCs) with aging | | | | | Flow cytometry, ELISA, migration and phagocytosis assays, real-time PCR, protein expression analysis | | DCs from elderly individuals showed reduced phagocytosis via macropinocytosis and endocytosis, impaired migration, and increased TNF-α and IL-6 secretion in response to LPS and ssRNA. Reduced phosphorylation of AKT suggested decreased activation of the PI3K pathway. Increased expression of phosphatase and tensin homolog (PTEN) may contribute to PI3K pathway dysregulation and altered innate immune responses in aging. | | | | | | Focuses on monocyte-derived DCs, does not assess in vivo relevance, limited to innate immune functions |
| Mathur et al., 2008 | | | | | Analyze age-related changes in eosinophil function and their implications for asthma | | | | | Comparison of eosinophil function in asthma patients aged 20–40 vs. 55–80 years (lung function, sputum analysis, in vitro eosinophil activity tests). | | Older adults showed reduced eosinophil degranulation in response to IL-5 (p=0.025) and lower superoxide production (p=0.097). No differences in lung function or sputum eosinophils. | | | | | | Limited to asthma patients; no in vivo functional validation; small sample size. |
| Jing et al., 2009 | | | | Investigate dendritic cell aging | | | | | Peripheral blood analysis, flow cytometry, cytokine secretion assays | | | Aging is associated with a decline in plasmacytoid dendritic cells (pDCs) and reduced IFN-α secretion in response to influenza virus. Toll-like receptor expression on pDCs is also reduced. In contrast, myeloid dendritic cells (mDCs) remain stable in healthy elderly individuals but are significantly depleted in frail elderly subjects | | | | | Cross-sectional study, limited sample size, requires longitudinal validation | |
| Muzzioli et al., 2009 | | | | Evaluate the effect of zinc on the development of CD34+ progenitors into NK cells in young and old individuals | | | | | CD34+ cells from peripheral blood of young and old healthy donors cultured with cytokines ± zinc supplementation; analysis of NK cell development and function | | | NK cell development and cytotoxic activity were lower in older donors; zinc supplementation increased cell numbers, NK cell percentage, and cytotoxicity in both age groups; GATA-3 expression was significantly upregulated with zinc | | | | | In vitro study; does not assess long-term effects of zinc supplementation in vivo | |
| Sokol et al., 2009 | | | | Investigate the role of basophils as antigen-presenting cells (APCs) in T helper type 2 (TH2) immune responses | | | | | In vitro and in vivo experiments using protease allergens to evaluate basophil antigen presentation | | | Basophils, rather than dendritic cells, were required and sufficient for TH2 differentiation in response to protease allergens | | | | | Study focused on specific allergens; potential differences in human immune responses require further validation | |
| Le Garff-Tavernier  et al., 2010 | | | | Characterize NK cell phenotypic and functional changes across life | | | | | Flow cytometry, NK cell subset analysis (CD56bright, CD56dim), receptor expression (KIR, LIR-1/ILT-2, NKG2A), IFN-γ production | | | Aging led to a decline in CD56bright NK cells and increased LIR-1/ILT-2 expression. NK function remained largely preserved, with IL-2 restoring activity in the very old. | | | | | Does not explore how these changes impact disease susceptibility or overall immune resilience | |
| Almeida-Oliveira et al., 2011 | | | | Investigate age-related changes in NK cell receptors from childhood to old age | | | | | Flow cytometry, NK cell subset analysis (CD56bright, CD56dim), receptor expression (KIRs, NCRs, C-type lectins), cytotoxicity assays | | | Elderly individuals showed decreased expression of activating receptors NKp30 and NKp46 on NK cells. KIR expression increased in the CD56bright subset. NKG2D expression was reduced in T cells of elderly subjects. Cytotoxic activity was preserved, suggesting a compensatory increase in CD56dim NK cells. KIR2DL5 and KIR2DS3 were significantly associated with old age. | | | | | Lacks functional studies on how these receptor changes impact overall immune responses to infections and malignancies | |
| Hearps et al., 2012 | | | | Investigate the effect of aging on monocyte subsets and function | | | | | Flow cytometry, cytokine assays, phagocytosis assays | | | Age associated with an increase in intermediate and nonclassical monocytes. Altered phenotype (e.g., increased CD11b, decreased CD38, CD62L, CD115). Elevated innate immune markers (CXCL10, neopterin, sCD163). Older monocytes showed impaired phagocytosis and higher intracellular TNF, suggesting dysregulated function. Significant sex differences in monocyte phenotype and immune markers. | | | | | Cross-sectional study, lacks longitudinal data, and does not address underlying mechanistic pathways | |
| Petrov et al., 2013 | | | | Investigate age-related changes in mast cells and eosinophils in human dermis | | | | | Quantitative analysis of mast cells and eosinophils in dermis of individuals across different age groups | | | Mast cell quantity increases with age; no age-related changes in eosinophils in dermis. Increase in mast cells correlated with decreased fibroblast proliferation (PCNA+ cells) | | | | | Limited to dermis; study only on mast cells and eosinophils, does not explore broader immune response changes | |
| Prakash et al., 2013 | | | | Investigate the effect of aging on DC function and interferon production in response to influenza | | | | | Monocyte-derived DCs, influenza virus stimulation, chromatin immunoprecipitation (ChIP) | | | Aged DCs showed reduced secretion of IFN-I and IFN-III in response to influenza, linked to age-associated chromatin modifications. Increased H3K9me3 repressor histone and decreased H3K4me3 activator histone at IFN promoter sites contributed to impaired IFN production. TNF-α promoter histone associations were unchanged. | | | | | Focuses only on monocyte-derived DCs, does not examine direct in vivo responses to influenza or broader immune cell interactions | |
| Hazeldine et al., 2014 | | | | Investigate age-related changes in neutrophil extracellular trap (NET) formation | | | | | Analysis of NET formation in neutrophils from young and older adults using TNF-α, LPS, IL-8, and PMA stimulation | | | LPS- and IL-8-induced NET formation declines with age due to reduced ROS generation; TNF-α priming increases NET formation in both age groups. No difference in PMA-induced NETs | | | | | Limited to in vitro assays; does not establish direct clinical consequences of reduced NET formation | |
| Jang et al., 2014 | | | | Investigate the role of reactive oxygen species (ROS) in collagen-induced platelet activation | | | | | Analysis of ROS production in platelets from wild-type and GPx1/catalase-deficient mice; measurements of SHP-2 oxidation and tyrosine phosphorylation signaling | | | Collagen-induced ROS generation oxidizes SHP-2, enhancing tyrosine phosphorylation of Syk, Vav1, Btk, and PLCγ2, promoting platelet activation and thrombus formation | | | | | Study conducted in mice; relevance to human platelet physiology needs further validation | |
| Qian et al., 2014 | | | | Investigate the effects of aging on TLR1 function and PMN bioenergetics | | | | | Analysis of PMN function in young (21–30 years) vs. older (>65 years) adults, including TLR1 expression, activation markers, cytokine production, and bioenergetics | | | Aging reduces TLR1 expression and downstream signaling, leading to lower CD11b/CD18 activation, IL-8 production, and p38 MAPK phosphorylation; PMN energy utilization is also impaired | | | | | Limited to in vitro studies; does not establish in vivo implications of reduced PMN bioenergetics | |
| Cini et al., 2015 | | | | Characterize differences in the resting platelet proteome and releasate between healthy children and adults | | | | | Proteomic analysis of platelets using Two-dimensional Differential In-Gel Electrophoresis and mass spectrometry; comparison between healthy children and adults | | | Significant differences in the expression of 9 proteins in resting platelets and 11 proteins in the platelet releasate; serotransferrin and thrombospondin-1 differentially regulated | | | | | Study limited to healthy individuals; functional consequences of these proteomic differences remain unclear | |
| Cowman et al., 2015 | | | | Examine the impact of age and gender on platelet function under arterial shear conditions | | | | | Blood from males (n = 53, ages 19-82) and females (n = 56, ages 21-70) perfused through parallel plate flow chambers coated with von Willebrand Factor (VWF); platelet translocation recorded via digital-image microscopy | | | Aging led to a decrease in platelet tracks, translocating platelets, and unstable platelet interactions with VWF; changes were more pronounced in women than in men | | | | | Study limited to in vitro conditions; potential physiological implications need further investigation | |
| Metcalf et al., 2015 | | | | Analyze age-related changes in innate immune responses after PRR stimulation | | | | | Transcriptional profiling, PBMC stimulation assays, flow cytometry, mixed lymphocyte reaction (MLR) | | | PBMCs from elderly individuals showed delayed and altered responses to TLR4, TLR7/8, and RIG-I agonists, with reduced production of TNF-α, IL-6, IL-1β, IFN-α, IFN-γ, CCL2, and CCL7. Monocyte and dendritic cell numbers were unchanged, but functional alterations included lower CD40+ monocytes and altered co-stimulatory protein expression (e.g., increased PD-L2 and B7-H4 on B cells). TLR-stimulated PBMCs from old individuals induced lower T-cell proliferation in an MLR assay. | | | | | Does not establish causal mechanisms, limited to ex vivo analyses of PBMCs | |
| Molony et al., 2017 | | | | | Investigate age-related impairments in antiviral interferon responses | | | | | Monocyte isolation, RIG-I signaling analysis, gene expression profiling | | Aging leads to defective type I interferon production in response to influenza A virus due to increased degradation of TRAF3 and reduced IRF8 expression, impairing antiviral defense | | | | | | tudy focused on monocytes, requires validation in other immune cell types and in vivo models |
| Campbell et al., 2018 | | | | | Investigate platelet-leukocyte interactions and cytokine synthesis in aging | | | | | Platelets and monocytes isolated from healthy younger (<45 years, n = 37) and older (≥65 years, n = 30) adults; incubation under autologous/nonautologous conditions; RNA sequencing of platelet transcriptome | | Monocytes from older adults, in the presence of autologous platelets, synthesized significantly higher IL-8 and MCP-1 levels; increased platelet granzyme A (GrmA) expression regulated cytokine production | | | | | | Study limited to healthy individuals; does not account for comorbidities that may influence platelet function and inflammation |
| Macal et al., 2018 | | | | | Investigate mechanisms sustaining exhausted plasmacytoid dendritic cells (pDCs) during chronic viral infection | | | | | Bone marrow and spleen analysis, flow cytometry, gene expression profiling | | Aging and chronic viral infection lead to dysfunction and self-renewal of pDCs via IFN-I and TLR7 signaling. Exhausted pDCs exhibit impaired IFN production, contributing to persistent infection and immune dysfunction | | | | | | Focus on murine model, need for validation in human systems and exploration of other innate immune cells |
| Pilkington et al., 2019 | | | | | Investigate how mast cells (MCs) contribute to human skin aging | | | | | Immunostaining, spatial morphometry, qPCR | | Aged skin showed a 40% increase in MCs, with reduced degranulation and altered interactions. VIP expression increased while substance P decreased, correlating with a threefold increase in VIP+ nerve fibers. | | | | | | Study focused on photoprotected skin, does not assess functional consequences of MC changes in aging-related skin conditions |
| Cao et al., 2020 | | | | | Assess complement system role in aging | | | | | Immunological and biochemical assays | | A biological age model using plasma peptides was developed, explaining 72.3% of the variation in chronological age. The model could be used for primary prevention and warrants further investigation | | | | | | Requires further validation in larger and more diverse populations |
| Wang et al., 2020 | Analyze laboratory tests and host immunity in COVID-19 patients with varying severity | | | | | Comparison of routine laboratory tests and immune cell profiles among 65 COVID-19 patients categorized as mild, severe, and extremely severe | | | | | | | Severe and extremely severe COVID-19 cases show increased inflammatory markers (e.g., IL-6, IL-10, D-dimer), reduced CD4+ and CD8+ T cell numbers, and altered activation of immune cells, suggesting dysregulated immune responses in critical cases | | | Small sample size, observational study, and lack of mechanistic insights into immune dysfunction | | |
| Zheng et al., 2020 | | | Investigate functional exhaustion of cytotoxic lymphocytes in COVID-19 patients | | | | | mmune profiling of NK cells and CTLs, biomarker analysis | | | Upregulation of NKG2A on NK and cytotoxic T cells correlates with functional exhaustion, reduced antiviral response, and disease progression in early COVID-19. Targeting NKG2A may help reinvigorate immune responses. | | | Limited sample size, short study duration, and need for further validation of NKG2A as a therapeutic target | | | | |
| Guirao et al., 2020 | | Evaluate the role of IL-6 as a biomarker for COVID-19 severity and mortality | | | | | IL-6 serum level measurement, correlation with disease severity and clinical outcomes | | | | High IL-6 levels correlate with severe pneumonia, need for mechanical ventilation, and increased mortality. A threshold of 35 pg/mL effectively predicts severe outcomes, and IL-6 receptor antagonist Tocilizumab was used in severe cases | | | | Small cohort (50 patients), observational study, and need for validation in larger populations | | | |
| Bernardi et al., 2020 | | Investigate sex differences in proatherogenic cytokine levels in 104 healthy adults | | | | | Cytokine measurement (IL-1β, IL-6, TNF-α), hormone analysis | | | | Men exhibited higher levels of IL-1β, IL-6, and TNF-α, which were associated with testosterone and testosterone/estradiol ratio. Sex differences contribute to higher cardiovascular risk in men | | | | Small sample size, focus on healthy adults, and limited mechanistic insights | | | |
| Mueller et al., 2020 | | | | | Investigate why COVID-19 affects older adults more severely | | | | | Examine why COVID-19 disproportionately affects older individuals | | Older adults have a 23-fold higher risk of COVID-19 mortality. Increased severity linked to impaired immune regulation, chronic inflammation, endothelial dysfunction, and comorbidities. Discusses aging biomarkers that could help predict disease severity and proposes interventions targeting immune resilience. | | | | | | Early COVID-19 study, later data may refine conclusions |
|  | |  | | | | |  | | | |  | | | |  | | | |
| Morrison et al., 2022 | | | Investigate SARS-CoV-2 transmissibility and the role of IL-13 in viral spread | | | | | Electron microscopy, gene expression analysis, biochemical assays | | | SARS-CoV-2 spreads via large viral clusters and massive epithelial cell shedding. IL-13 reduces viral entry, replication, and cell-to-cell transmission, suggesting a protective role in COVID-19 | | | In vitro study, requires in vivo validation and further exploration of IL-13's therapeutic potential | | | | |
| Sabbatini et al., 2022 | | | Investigate the impact of aging on NETs' efficacy | | | | | Neutrophils from elderly (>65 years) and adults (20-50 years) were stimulated with LPS to induce NETs; NETs were quantified, analyzed for DNA size, and tested for bactericidal activity and keratinocyte proliferation stimulation | | | Elderly subjects produced more NETs than adults, but these NETs had reduced bactericidal capacity and impaired stimulation of keratinocyte proliferation. NETs from elderly had larger DNA structures. | | | Limited sample size, in vitro study, lacks in vivo validation of NETs functionality in elderly subjects. | | | | |
| Quin et al., 2024 | | | Investigate sex differences in intestinal barrier function and monocyte-driven inflamm-aging | | | | | Flow cytometry, cytokine assays, animal models, mediation analysis | | | Females exhibited higher intestinal permeability, increased circulating bacterial products (LPS, kynurenine), and higher markers of inflamm-aging (TNF, CRP). Inflammation was found to precede the increase in bacterial products, and sex differences were observed in monocyte levels and inflammatory responses. The TNF to IL-10 ratio increased with age in females, indicating altered pro-inflammatory to anti-inflammatory balance. Males had higher absolute TNF but no age-related change | | | Limited to observational and mediation analysis, does not conclusively identify causality or mechanisms | | | | |
| Cui et al., 2024 | | | Assess immune signature and phagocytosis of circulating DC subsets during aging | | | | | Multi-color flow cytometry, magnetic bead sorting, co-culture with polystyrene beads | | | Aging was associated with decreased circulating cDC1, cDC2, and CD32+cDC2 percentages. Phagocytic function of cDC2 declined with age. Established reference ranges for DC subsets based on age and sex. | | | Cross-sectional study, lacks functional assays on antigen presentation and adaptive immune interactions | | | | |
|  | | |  | | | | |  | | |  | | |  | | | | |
| Law et al., 2005 | | | To investigate how SARS-CoV affects human monocyte-derived dendritic cells (DCs) and contributes to immune evasion | | | | | Infected immature and mature human monocyte-derived DCs with SARS-CoV and analyzed viral replication, cytokine production, and cell maturation using electron microscopy, immunofluorescence, and cytopathic assays | | | SARS-CoV infects DCs but fails to complete viral replication. Infected DCs show low antiviral cytokine production (IFN-α, IFN-β, IFN-γ, IL-12p40) but upregulate pro-inflammatory cytokines (TNF-α, IL-6) and chemokines (MIP-1α, RANTES, IP-10, MCP-1). The absence of an antiviral response alongside chemokine upregulation suggests a mechanism of immune evasion. | | | Study is based on in vitro DC models and may not fully capture in vivo immune dynamics. The role of other immune cells and systemic responses needs further exploration | | | | |
| Josset et al., 2013 | | | To compare the host response to infection with HCoV-EMC (novel human coronavirus) and SARS-CoV in human lung epithelial cells. | | | | | Transcriptomic analysis of Calu-3 cells infected with either HCoV-EMC or SARS-CoV. Genes dysregulated during infection were identified and potential antiviral compounds were predicted through computational screening. | | | HCoV-EMC induced a more extensive dysregulation of the host transcriptome than SARS-CoV, including down-regulation of antigen presentation genes. Both viruses activated pattern recognition receptors and IL-17 pathways similarly. A unique set of genes was identified, allowing the prediction of potential antiviral compounds, including kinase inhibitors and glucocorticoids. | | | The study is based on a cell culture model, which may not fully reflect in vivo host responses. Further studies in animal models or humans are needed to validate these findings. | | | | |
| Muthana et al., 2016 | | | To evaluate the effects of age, race, gender, and blood type on anti-glycan antibody profiles in human serum. | | | | | Serum anti-glycan antibody levels were measured in 135 healthy subjects, analyzing IgG and IgM signals to various glycan antigens. | | | The study found that IgG and IgM antibodies to blood group antigens strongly correlated with blood type. IgM signals decreased with age, but no significant change in IgG levels was observed. There were also significant race-related correlations in IgG levels to certain LacNAc-containing glycans. | | | The study was conducted on a limited cohort of healthy individuals, and the findings may not directly apply to diseased populations. Further research in clinical settings is needed to confirm these results. | | | | |
| Molony et al., 2017 | | | To investigate the impact of aging on RIG-I signaling and type I interferon (IFN) production in monocytes, and its implications for respiratory influenza A virus (IAV) infections. | | | | | Monocytes from younger and older adults were stimulated with IAV RNA to assess type I IFN production. The expression of signaling proteins and transcription factors was measured, and the role of IRF8 and TRAF3 in IFN induction was analyzed. | | | Aging impairs both primary and secondary RIG-I signaling in monocytes, reducing IFN production. The defect is associated with decreased levels of TRAF3 and impaired activation of the transcription factor IRF8. Restoration of IRF8 expression in older adults' monocytes restores IFN responses. | | | The study focuses on in vitro experiments with human monocytes, and further in vivo studies are needed to confirm these findings in the context of influenza infection. | | | | |
| Conti et al., 2020 | | | To explore the role of mast cells (MCs) and histamine in the inflammatory response and cytokine storm associated with SARS-CoV-2 infection. | | | | | Mechanistic hypothesis discussing the activation of MCs by SARS-CoV-2 and their role in the production of histamine and cytokines such as IL-1 and IL-6 | | | MC activation leads to histamine release, which increases IL-1 levels, amplifying the inflammatory response in the lungs of COVID-19 patients. Histamine receptor antagonists may help mitigate IL-6 production and inflammation. | | | The study is based on previous literature and mechanistic hypotheses rather than direct experimental data. Further in vivo and clinical studies are needed to validate the proposed mechanisms. | | | | |
| Hadjadj et al., 2020 | | | To investigate the role of type I interferon (IFN) responses in severe COVID-19 cases. | | | | | Analyzed peripheral blood immune cells from severe and critical COVID-19 patients, measuring IFN levels and inflammatory cytokines (IL-6, TNF-α). Compared to mild cases. | | | Severe COVID-19 patients showed decreased type I IFN responses and increased IL-6 and TNF-α-driven inflammation. Suggests an imbalance where systemic IFN responses are suppressed while local IFN expression in the lungs may contribute to pathology. | | | The study focuses on peripheral blood cells and does not fully capture IFN responses at the tissue level. Causality between IFN dysregulation and disease severity needs further validation. | | | | |
| Hottz et al., 2020 | | | Investigate platelet activation and platelet-monocyte interactions in severe COVID-19 | | | | | Blood analysis from COVID-19 patients; platelet activation and monocyte tissue factor (TF) expression assays; in vitro platelet stimulation experiments | | | Increased platelet activation and platelet-monocyte aggregates in severe COVID-19; strong association with TF expression and coagulation markers (fibrinogen, D-dimers); platelet-induced TF expression inhibited by P-selectin neutralization or integrin αIIb/β3 blocking | | | Observational study; limited to ICU patients; lacks long-term follow-up | | | | |
| Wang et al., 2020 | | | To compare routine laboratory tests and host immunity in COVID-19 patients with different severity of illness after patient admission. | | | | | A total of 65 SARS-CoV-2–positive patients were classified as having mild, severe, and extremely severe illness. Routine laboratory tests (e.g., ferritin, lactate dehydrogenase, D-dimer), immune cell counts, and activation markers were measured. | | | Severe and extremely severe patients showed increased ferritin, lactate dehydrogenase, and D-dimer levels. T cell (CD4+, CD8+) and B cell counts were gradually decreased with increasing severity. Activation markers (HLA-DR, CD45RO) increased in severe and extremely severe cases. Natural Tregs were decreased in extremely severe patients. Cytokines (IL-2R, IL-6, IL-10) were elevated in severe cases. CD8+ and CD4+ T cells produced higher levels of IFN-γ in severe and extremely severe patients. DC and B cell activation was decreased in extremely severe cases. | | | The study primarily focuses on immune responses and laboratory tests; further analysis of long-term immune changes and outcomes was not conducted. | | | | |
| Zaid et al., 2020 | | | Evaluate platelet involvement in inflammation and thrombosis in COVID-19 | | | | | Blood samples from 115 COVID-19 patients (71 nonsevere, 44 severe) analyzed for platelet-associated SARS-CoV-2 RNA, cytokine levels, and platelet activation markers | | | Platelets harbored SARS-CoV-2 RNA and were hyperactivated in COVID-19 patients; platelet activation contributed to cytokine release and thrombotic risk | | | Study did not explore the long-term consequences of platelet hyperactivation in COVID-19 survivors | | | | |
| Zhou et al., 2020 | | | To investigate the impact of acute SARS-CoV-2 infection on immune cell populations and adaptive immune responses. | | | | | Analysis of immune cell populations in 17 acute and 24 convalescent COVID-19 patients using flow cytometry and functional assays | | | Acute SARS-CoV-2 infection causes broad immune cell reduction, including dendritic cells (DCs), T cells, NK cells, and monocytes. DCs are functionally impaired, with altered cDC:pDC ratios in severe cases. Neutralizing antibodies are rapidly generated, but RBD- and NP-specific T cell responses are delayed and skewed toward CD4 rather than CD8 T cells. | | | Study is limited by a relatively small sample size and lack of longitudinal tracking beyond early convalescence. Further research is needed to confirm findings in larger cohorts. | | | | |
| Avila-Nava et al., 2021 | | | To assess the serum IL-6 levels as a potential biomarker for mortality among COVID-19 patients | | | | | A cohort study involving 38 adults (28 men, 10 women) in the Regional High Specialty Hospital of Yucatan. Demographic, clinical, and biochemistry data were collected, and serum IL-6 levels were measured by immunoassay | | | Mortality rate was 36.84%. IL-6 levels were significantly higher in non-surviving patients. The average age of non-survivors was significantly higher than survivors. IL-6 correlated with lymphocyte count, LDH, CRP, and procalcitonin. The optimal IL-6 cutoff value was 30.95 pg/mL, with high sensitivity and specificity for mortality prediction. | | | The study only included 38 patients, which limits the generalizability of findings. A larger sample size would strengthen the results. | | | | |
| Cazzaniga et al., 2021 | | | To evaluate the role of eosinopenia as a prognostic marker in COVID-19 pneumonia. | | | | | Retrospective analysis of 107 COVID-19 pneumonia patients. Compared clinical outcomes of patients with and without absolute eosinopenia. Logistic regression used to assess associations with mortality, intensive respiratory support, and hospital discharge | | | Absolute eosinopenia was associated with higher need for intensive respiratory support (49.3% vs 13.3%, P < .001), higher mortality (30.6% vs 6.2%, P = .006), and lower hospital discharge rate (28% vs 65.6%, P < .001). Identified as an independent predictor of poor prognosis. | | | Retrospective, single-center study; potential confounding factors not fully addressed; no external validation of findings. | | | | |
| Valle Martins et al., 2021 | | | To investigate the circulating levels of angiotensin-(1–7) and angiotensin II in severely ill COVID-19 patients and assess a possible dysregulation in the renin–angiotensin system. | | | | | Letter reporting the unexpected increase of angiotensin-(1–7) and decrease of angiotensin II in COVID-19 patients, suggesting a shift in the renin–angiotensin system. | | | Angiotensin-(1–7) levels were found to be significantly increased in severely ill COVID-19 patients, while angiotensin II levels decreased, indicating a dysregulation of the renin–angiotensin system. The study hypothesizes that Ang-(1–7) could improve clinical outcomes in COVID-19 patients. | | | The study is a letter and does not provide detailed clinical trial data. Further clinical trials are needed to confirm these findings. | | | | |
| Colicchia et al., 2022 | | | Investigate the role of S100A8/A9 in platelet function and thrombosis, particularly in COVID-19 | | | | | Plasma and lung autopsy analysis from COVID-19 patients; in vitro platelet adhesion and activation assays; genetic and pharmacologic inhibition studies | | | Increased S100A8/A9 levels in COVID-19 correlated with poor outcomes; S100A8/A9 induced procoagulant platelet formation via GPIbα; blocking GPIbα prevented S100A8/A9-mediated effects on platelets | | | Mechanistic study; limited clinical data; potential species differences in mouse models | | | | |
| Ito et al., 2023 | | | To investigate the association between eosinopenia and adverse outcomes in COVID-19 patients. | | | | | Retrospective study on 125 hospitalized COVID-19 patients in Japan, analyzing eosinophil count (Eos = 0 vs. Eos > 0) and clinical outcomes (ICU admission, disease severity, and mortality). | | | Eosinopenia (Eos = 0) was significantly associated with ICU admission (OR 4.89, p = 0.009) and increased disease severity (OR 4.20, p = 0.02), but not with 30-day mortality. Other predictors of mortality included age, diabetes, and lactate dehydrogenase levels | | | Single-center, retrospective study; relatively small sample size; lack of mechanistic insights into the role of eosinophils in COVID-19 severity | | | | |
| Xu et al., 2024 | | | To investigate the impact of SARS-CoV-2 envelope (E) protein on airway epithelial barrier function, Cl− transport, and airway inflammation. | | | | | Study analyzing the effect of SARS-CoV-2 E protein on airway epithelial cells. The expression of tight junction proteins, activation of Toll-like receptors (TLR) 2/4, and signaling pathways related to inflammation were assessed | | | E protein down-regulated tight junctional proteins, disrupting the airway epithelial barrier. It activated TLR2/4 and JNK signaling, leading to increased intracellular Cl− concentration. This elevation in Cl− contributed to heightened airway inflammation via SGK1 phosphorylation. Blocking SGK1 or PDE4 alleviated the inflammation induced by E protein. | | | The study is based on in vitro experiments, which may not fully replicate the complexity of in vivo conditions in humans. Further animal or clinical studies are needed to confirm these findings. | | | | |
| Elnagdy et al., 2024 | | | To investigate the genetic association between ACE2 and TMPRSS2 polymorphisms and COVID-19 severity. | | | | | Cross-sectional study of 317 Egyptian patients with mild and severe COVID-19, genotyping ACE2 rs2285666 and TMPRSS2 rs12329760 polymorphisms using TaqMan real-time PCR. | | | No significant association between ACE2 rs2285666 polymorphism and COVID-19 severity. However, TMPRSS2 rs12329760 minor T allele and CT/TT genotypes were associated with a reduced likelihood of severe COVID-19. | | | The study was conducted in a single center with a specific demographic, limiting generalizability. Further research across diverse populations and considering other genetic factors is needed. | | | | |

**Table S2.** Adaptive Immunity and Aging: Summary of Main Findings on B and T Lymphocytes

| Study | | Objective | | Methods | | Main Findings | | Limitations |
| --- | --- | --- | --- | --- | --- | --- | --- | --- |
| Karanfilov et al., 1999 | Investigate if age-related changes in Th1/Th2 cytokine production are linked to altered naive (CD45RA+) and memory (CD45RO+) T cell frequencies | | T cells from young and elderly individuals were stimulated with anti-CD3ε + PMA; cytokine production was measured and correlated with T cell subset distribution via flow cytometry | | Elderly individuals showed decreased IL-2, IFN-γ (Th1) and IL-4 (Th2) production; no significant shift in Th1/Th2 balance; naive T cells declined while memory T cells increased, but cytokine defects were independent of these changes | | Does not establish the molecular cause of cytokine production decline; limited to ex vivo analysis | |
| Bandres et al., 2000 | Investigate the correlation between aging, IFN-γ production, and specific CD8+ T cell subpopulations | | Flow cytometry analysis of intracellular IFN-γ and IL-4 in CD4+ and CD8+ T cells from healthy donors (17-62 years); multivariate analysis to assess aging vs. chronic antigenic exposure | | IFN-γ production increased with age, particularly in CD8+high T cells; correlated with CD8+highCD28−CD57+ subset, which is expanded in older individuals | | Limited age range (up to 62 years); does not establish causation between subset expansion and immune function changes | |
| Naylor et al., 2005 | Examine the effect of aging on T cell generation and TCR diversity | | Analysis of TCR excision circles (TREC), Ki67(+) cycling CD4 T cells, and TCR β-chain diversity in young (~25 years), middle-aged (~60 years), and elderly (~75 years) adults | | Thymic output (TREC) declines >95% by age 60; homeostatic proliferation doubles after 70; CD4 TCR diversity collapses from ~2×10⁷ to ~200,000 unique β-chains, indicating major T cell loss | | Cross-sectional study design; does not track individual immune changes over time | |
| Brandl et al., 2011 | Investigate the effects of oxidative stress on mesenchymal stem cells (MSCs) and their senescence | | Exposure of MSCs to acute and prolonged oxidative stress using hydrogen peroxide; analysis of proliferation, morphology, telomere length, and gene expression | | Acute oxidative stress reduced proliferation, induced senescence-like features, and accelerated telomere attrition; MSCs showed increased tolerance compared to fibroblasts and chondrocytes, but aged MSCs had impaired stress tolerance | | In vitro study; limited translation to in vivo conditions; does not explore potential protective mechanisms | |
| Hao et al., 2011 | Identify and characterize a B-cell subset that accumulates with age | | Analysis of B-cell subsets in aged mice, response to BCR/CD40 and TLR stimulation, cytokine production, BLyS receptor expression, and antigen presentation | | Discovered age-associated B cells (ABCs) that accumulate with age, are refractory to BCR/CD40 stimulation, but respond to TLR9/TLR7; produce IL-10 and IL-4, present antigen effectively, and favor TH17 polarization | | Mechanisms of ABC generation require further investigation | |
| Knippenberg et al., 2011 | Assess B cell subsets, including Bregs, in RRMS patients during stable and active disease | | Flow cytometry analysis of B cell subsets in RRMS patients and healthy controls; correlation with vitamin D status | | Reduced memory B cells and B_regs_; relapse specifically reduces naïve B_regs_; no correlation with vitamin D levels | | Functional impact of vitamin D on B_regs_ not ruled out; small sample size | |
| Clark et al., 2012 | Investigate MHC-II antigen presentation by B cells | | T cell hybridoma system, flow cytometry, antigen presentation assays | | Antigen presentation via BCR-mediated endocytosis was heterogeneous in older individuals, with some exhibiting impaired processing. Peptide presentation remained unchanged. HLA-DR and IgM expression on B cells and monocytes were not significantly different between young and old subjects. Adhesion molecules CD54 and CD58 showed no differences between groups. | | Small sample size, lacks mechanistic insights into causes of variability in antigen presentation | |
| Bektas et al., 2013 | Investigate age-associated changes in TCR-inducible gene expression in human CD4+ T cells | | Analysis of gene expression in CD4+ T cells from young and old individuals, focusing on NF-κB target genes | | NF-κB target genes are not sustainedly induced in older individuals, except for pro-inflammatory genes (IL-1, IL-6) | | Does not address causality; limited to transcriptomic analysis without functional validation | |
| Frasca et al., 2016 | Investigate memory B cell generation and antibody response to influenza vaccination in the elderly | | Repeated influenza vaccinations in young and elderly individuals, measuring IgG vaccine-specific memory B cells and serum antibody response | | Memory B cell generation is maintained, but the antibody response is impaired in the elderly after repeated vaccinations, with reduced serum titers and reduced differentiation of memory B cells to plasma cells | | Transcription factors associated with plasma cell differentiation were reduced in the elderly | |
|  |  | |  | |  | |  | |
| Ciocca et al., 2021 | Investigate the evolution of human memory B cells across different age groups | | Flow cytometry analysis of peripheral blood from children (4-18 years), young adults (23-60 years), and elderly (65-91 years); ELISA for antibody secretion after T-independent stimulation | | Elderly individuals show a reduction in CD27dull memory B cells, crucial for bridging innate and adaptive immunity; memory B cells are mostly CD27bright; reduced IgM and IgA production after CpG stimulation | | Cross-sectional study design: functional implications of CD27dull B cell reduction require further research | |
| Glass et al., 2022 | Characterize the immunophenotype and induction of IL-10-producing regulatory B cells (B_regs_) | | Mass cytometry to quantify cytokine production and immunophenotype of human peripheral B cells under various stimuli | | IL-10+ B cells arise from multiple B cell subsets, lacking a unique phenotype; many co-express IL-6 and TNFα | | No single marker for prospective isolation of IL-10+ B cells; functional role in immune regulation requires further study | |
| Andreu-Sánchez et al., 2024 | Explore relations between microbial exposures and biological aging, cell composition, and inflammation | | Profiling of antibody-binding repertoire to 2,815 microbial, viral, and environmental peptides in a cohort of 1,443 participants | | Immune responses to CMV, rhinovirus, and gut bacteria are linked to telomere length; CMV infection affects the transcriptional landscape of CD8 and CD4 T-cells | | No specific experimental details provided; study scope unclear | |
|  |  | |  | |  | |  | |
| Haynes, 2020 | Examine T-cell aging and immune decline | | Review of T-cell subsets in aging | | Decline in naïve T cells, expansion of memory T cells, and reduced vaccine efficacy | | Lacks experimental validation | |
| Guan et al., 2024 | Analyze CD28 expression in aging T cells | | Flow cytometry and functional assays | | Decreased CD28 expression reduces costimulatory signaling, leading to impaired T-cell responses | | No interventional studies | |
| Fujimori & Ohigashi, 2024 | Examine thymic involution and T-cell production | | Histological and immunological analysis | | Decreased thymic output leads to reduced naïve T-cell generation with age | | Limited sample size | |
| Nicoli et al., 2020 | Assess T-cell responsiveness in COVID-19 | | Serological and T-cell activation assays | | Age-related decline in T-cell activation contributes to severe COVID-19 outcomes | | Focused mainly on COVID-19 patients | |
| MacIver et al., 2013 | Investigate metabolic regulation of T cells | | Metabolic and functional assays | | Aging-associated metabolic shifts impair T-cell function | | Lacks direct aging intervention studies | |

**Table S3.** Aging and COVID-19: Summary of Main Findings

| **Aspect** | | **Young Individuals** | **Elderly Individuals** | | |
| --- | --- | --- | --- | --- | --- |
| **ACE2 Expression** | Higher ACE2 expression in tissues | | | Reduced ACE2 expression with aging | |
| **Effects on Infection** | Higher ACE2 availability may facilitate viral entry but with fewer systemic damages | | | Lower ACE2 levels lead to reduced ability to counterbalance inflammation, increasing tissue damage risk | |
| **Inflammation and Immune Response** | Faster and more effective immune response | | | | Chronic inflammation ("inflammaging") and less efficient immune response |
| **Lung Damage** | Lower risk of severe lung damage | | | | Higher susceptibility to lung damage due to inflammation dysregulation |
| **Risk of Complications** | Lower risk of severe COVID-19 forms | | | | Higher risk of pneumonia, thrombosis, and multi-organ failure |
| **Pro-inflammatory Cytokines (IL-6, TNF-α, IL-1β)** | Moderate increase, controlled response | | | | Excessive production, leading to hyperinflammation |
| **Anti-inflammatory Cytokines (IL-10, TGF-β)** | Balanced response, effective resolution of inflammation | | | | Reduced or delayed response, leading to prolonged inflammation |
| **Cytokine Storm Susceptibility** | Lower risk due to effective immune regulation | | | | Higher risk of cytokine storm, contributing to severe COVID-19 |
| **Interferon Response (IFN-I, IFN-III)** | Strong and rapid antiviral response | | | | Delayed or weakened interferon response, allowing higher viral replication |
| **Immune Cell Recruitment** | Efficient recruitment of immune cells to infection site | | | | Impaired recruitment, leading to ineffective viral clearance |
| **Neutrophil Activation** | Balanced activation, effective pathogen clearance | | | | Overactivation, leading to tissue damage and excessive inflammation |
| **Macrophage Function** | Pro-inflammatory and anti-inflammatory balance | | | | Skewed towards pro-inflammatory state, exacerbating tissue damage |
| **Dendritic Cell Function** | Effective antigen presentation, robust adaptive immune activation | | | | Reduced antigen-presenting capacity, weak adaptive immune response |
| **Monocyte Response** | Well-regulated monocyte activation and differentiation | | | | Dysregulated monocyte activity, contributing to chronic inflammation |
| **Inflammation Resolution** | Timely resolution of inflammation, preventing excessive tissue damage | | | | Impaired resolution, leading to prolonged inflammation and tissue injury |
| **Treg Function in Inflammation** | Effective at suppressing excessive inflammation and preventing tissue damage. | | | | In older adults, Tregs exhibit enhanced immunosuppressive activity but may fail to adequately suppress Th17 cells, leading to increased inflammation |
| **Treg Cytokine Secretion** | Tregs efficiently secrete immunosuppressive cytokines (e.g., IL-10, TGF-β) | | | | Production of anti-inflammatory cytokines by Tregs is diminished |
| **Treg and COVID-19** | Tregs may help limit severe inflammation in young people with COVID-19. | | | | Decreased Treg activity may contribute to higher severity of COVID-19 and poor clinical outcomes |
| **CD4 T Cell Function** | Balanced effector and regulatory roles, allowing effective immune response | | | | Imbalance between effector and regulatory roles, leading to potential dysregulation in response to COVID-19 infections |
| **Thymic Output** | Higher thymic activity, producing more naïve T cells | | | | Significant decline in thymic function after 40-50 years, leading to reduced naïve T cell production |
| **TCR Diversity** | Higher TCR diversity, allowing better response to novel pathogens | | | | Reduced TCR diversity due to antigen-independent proliferation of memory T cells |
| **Naïve T Cell Proportion** | Higher proportion of naïve T cells, enabling stronger responses to new infections | | | | Lower proportion of naïve T cells, replaced by antigen-experienced cells, reducing adaptability |
| **Response to Novel Pathogens** | Faster and more effective adaptive immune response | | | | Slower, lower-affinity response, increasing vulnerability to infections like COVID-19 |
| **Th1 Cells** | Th1 response effectively clears infection while avoiding excessive inflammation | | | | Initial Th2 predominance followed by a rapid Th1 shift can lead to cytokine storm and tissue damage |
| **Th2 Cells** | Anti-inflammatory Th2 response helps regulate excessive Th1 activation | | | | Predominant Th2 response early in infection allows viral replication before an uncontrolled Th1 response occurs |
| **Th1/Th2 Balance in COVID-19** | Balanced Th1/Th2 response, allowing efficient pathogen elimination with minimal host damage | | | | Th2 responses are upregulated initially, leading to higher susceptibility to infections like influenza and tuberculosis |
| **Th17 Cell Levels** | Lower levels of Th17 cells and IL-17 cytokine production | | | | Increased Th17 cell presence and IL-17 production, especially in individuals with inflammatory diseases |
| **IL-17 Secretion** | IL-17 plays a role in immune defense but is controlled to prevent excessive damage | | | | High IL-17 levels correlate with more severe disease, ARDS risk, and complement cascade activation |
| **CD8 T Cell Levels** | Higher and more sustained CD8 T cell counts post-infection. Majority of recovered patients develop a CD8 T cell response | | | | Lower baseline CD8 T cell counts, with further reductions post-infection. Weaker and less sustained CD8 response, leading to prolonged infection and worse outcomes |
| **CD8 T Cell Function** | More effective in recognizing and eliminating infected cells | | | | Reduced function and responsiveness, contributing to higher viral titers |
| **CD8 T Cell Exhaustion** | PD-1 and Tim-3 markers upregulated in severe cases but still functional in mild cases | | | | Increased expression of exhaustion markers (PD-1, Tim-3, NKG2A) leading to impaired proliferation and cytokine production |
| **Cytotoxic Activity** | Granzyme B and perforin production help control infection | | | | Upregulated cytotoxic granules, but overall lower cytotoxic efficiency due to exhaustion |
| **B Cell** | Faster and stronger antibody response | | | | Reduced production due to bone marrow degeneration Slower and weaker response, increasing susceptibility to COVID-19 |
| **Antibody Production** | Rapid and high-affinity antibody response to new pathogens. Higher rates, leading to better adaptation to new infections | | | | Delayed production of antibodies with lower affinity to new antigens, limiting the diversity and effectiveness of antibody responses |
| **Memory T and B Cell** | Efficient formation of antigen-specific memory T and B cells. Memory cells contribute to faster immune response | | | | Memory cells accumulate but may show dysfunction. Increased numbers in aged tissues, but with aberrant proinflammatory phenotypes and impacting infection control |
| **DPP4 and SARS-CoV-2** | Limited potential interaction | | | | DPP4 upregulation may contribute to severe COVID-19 outcomes­ |
